# Moving for Diversity or Moving for the Kids? The Micro-Dynamics of Residential Relocations During Family Formation of Immigrants and Natives

**DOI:** 10.3389/fsoc.2020.538946

**Published:** 2020-10-26

**Authors:** Michael Windzio, Mareike Oeltjen, Alice Blanksma

**Affiliations:** SOCIUM, Research Center on Inequality and Social Policy, University of Bremen, Bremen, Germany

**Keywords:** residential relocation, family formation, ethnic colonies, event history analysis, local schools

## Abstract

Family formation is a crucial event in the life course and generates a major part of residential relocations. After family formation, neighborhoods become re-evaluated, now as contexts for children's development and socialization. We argue that the perceived or assumed quality of schools and neighborhoods is an important condition of choosing a destination. However, as the literature on “ethnic colonies” and immigrant-native residential segregation suggests, immigrants differ from natives in their neighborhood preferences and relocation patterns. If relocations of migrant and native families to particular destinations do indeed occur basically during family formation and family enhancement, and if they are at the same time outcomes of different preferences, the micro-dynamics of young families' adaptation of housing conditions might have a considerable impact on segregation. Results of our ordered Heckman probit and event history models show that on the one hand, immigrants and natives tend to different evaluations of characteristics in their neighborhoods. Particularly respondents of Turkish, Arabic or African origin highly appreciate living nearby a house of worship and also with many Non-Germans. On the other hand, our analysis of how these evaluations transform into residential relocations did not show any differences between immigrants and natives. Results thus suggest that evaluations or preferences during family formation do not trigger relocations which result in “ethnic colonies” at the macro level.

## Introduction

Residential segregation is often analyzed using aggregate data and descriptive measures, e.g., the Duncan Index or indices of exposure or isolation. However, trends of segregation at the level of cities or other spatial units result from micro-processes, namely from relocation decisions. This micro-foundation of segregation is taken for granted since Th. Schelling introduced his model of “micromotives and macrobehavior” (Schelling, 1978), but there are only few studies analyzing these micro-processes empirically in a longitudinal perspective (Crowder, 2000; Quillian, 2002; Lersch, 2013).

Following the early work of Rossi (1954), we assume that many residential relocations are adaptations to events of family formation and family extension during the life course. Such events trigger a considerable part of residential relocations. Neighborhoods become re-evaluated after family formation, now as contexts for children's socialization and development. We argue that the perceived quality of schools and neighborhoods is an important condition of choosing a particular destination.

It is yet an open question whether relocations during family formation of migrant and native families are influenced by the same preferences and neighborhood evaluations. Following the literature on “ethnic colonies” and immigrant-native residential segregation, it can be concluded that immigrants differ from natives in their neighborhood preferences and relocation patterns (Lersch, 2013). If relocations of migrant and native families do indeed occur mainly during family formation and family enhancement (Rossi, 1954), and if relocations are at the same time outcomes of different preferences, the micro-dynamics of young families' adaptation of housing conditions might have a considerable impact on segregation.

To find out whether natives and migrants differ in their residential preferences, we analyse in a first step whether migrant families value indicators of social embeddedness and neighborhood diversity higher than non-migrant families do. Our neighborhood indicators are subjective evaluations of proximity to relatives, perceived diversity, proximity to religious institutions and proximity to a desired primary school.

Instead of primarily focusing on socio-economic factors, such as the mismatch of household income and housing prices, we test in a second step whether indicators of “ethnic social capital” do better explain residential moves of migrants during family formation than the proximity to the desired primary school. If the educational infrastructure in the neighborhood was more important than the local “ethnic social capital,” immigrants' relocation patterns would be more in line with “moving for the kids” (Goyette et al., 2014) then “moving for diversity.” Hence, the aim of our paper is to obtain a better understanding of relocation decisions of native and migrant families, which leads to a better understanding of the underlying “micromotives” (Schelling, 1978) of residential segregation.

In the empirical part of our paper, we use unique data from the “Moving for the Kids” project (funded by the DFG, German Research Foundation, grant no. 318053447), in which more than 6,000 parents of 2nd and 3rd grade kids in elementary schools where interviewed about neighborhood perception and past relocations in a self-administered survey. To test whether evaluations of neighborhoods differ between immigrants and natives, we use an ordered probit-regression, which controls for self-selection into a respective neighborhood-condition. Furthermore, we apply event history models to test whether the evaluation of neighborhood characteristics related to diversity and social embeddedness influence residential relocations, how relevant these factors are compared with the educational infrastructure and whether the effects differ between immigrant and non-immigrant families.

The structure of the paper is as follows: In section Theory and Research on Ethnic Residential Preferences, Relocations, and Segregation, we will start with a short overview on theory and research on residential segregation with particular reference to residential preferences of migrants. In the third part, we will give a short introduction into our measurements and statistical methods. Empirical results will be presented in section Result, which is divided into two parts: First, we analyse whether migrant families evaluate indicators of social embeddedness and neighborhood diversity higher than non-migrant families. Second, we test whether migrant families tend to different relocation decisions, given their evaluations. In the fifth and last section, we will summarize our findings and draw a conclusion regarding our research questions.

## Theory and Research on Ethnic Residential Preferences, Relocations and Segregation

According to Th. Schellings theoretical model (Schelling, 1978), the interdependence of preferences at the micro-level and inherent system dynamics at the macro-level tends to perfect segregation if (random) changes in ethnic neighborhood composition trigger cascades of relocations to neighborhoods where households can realize their preference of not being in a *small* local minority. As an unintended result of micro-level behavior (Boudon, 1981), the macro-level outcome of strong segregation can be regarded as a “perverse effect” (van Parijs, 1982), which means that the outcome is in sharp contrast to the rather inclusive “taste for diversity” (Dancygier and Laitin, 2014) in both groups.

In contrast to Schelling's “taste-for-diversity” assumption, early Chicago School sociologists explained segregation patterns with socio-economic inequalities, processes and practices of exclusion, but also with own-ethnic preferences. Ethnic communities can provide social support especially for newly arriving immigrants. Often, members of the ethnic community already assist in planning the emigration (Park et al., 1967). Since immigrants' “social capital” (Portes, 1998) usually emerges at the local level (Windzio and Trommer, 2019), they tend to spatial concentration. In the long run, growing ethnic communities reduce incentives to invest into receiving-context cultural and social capital also in the 2nd generation (Esser, 2010).

Concerns over potentially disintegrative effects of rigid ethnic-cultural boundaries crystallize in the terms “*ethnic colonies*” (Taeuber and Taeuber, 1964) or “*parallel societies*” (Heitmeyer, 1996), which highlight the separation of ethnic communities from the majority population and describe a situation similar to “institutional completeness” (Breton, 1964). Ethnic groups do not only tend to spatial clustering and dense strong-tie networks within their own communities, but they also create their own ethnic institutions and organizations, namely businesses, schools, or even legal institutions for different religious groups (Tibi, 2002, p. 46). Regarding this mode of ethnic integration, P. Collier's distinction between *emigrants* and *settlers* (Collier, 2013) challenges optimistic views about multiculturalism. While emigrants are willing to change group membership and adapt to norms of the receiving group (Taft, 1957), settlers bring their own institutions, norms and taken-for-granted knowledge and try to install their own “social model” in the acquired territory (Collier, 2013, p. 92). Social network ties within “settler” communities create “bonding” instead of “bridging” social capital (Putnam, 2000), and thereby reinforce ethnic boundaries (Wimmer, 2013; Windzio, 2018). Within the liberal-democratic framework of most host countries, institutions of an ethnic colony develop a new self-understanding and perform many more functions than they would in the country of origin. For example, mosques are no longer just spiritual places, but become important places of self-help and socio-cultural exchange (Ceylan, 2006, p. 252).

Describing “ethnic colonies” in total as “parallel societies” is, according to Ceylan (2006, p. 256), inappropriate because of their different social segments with various including and excluding functions that satisfy the social, cultural and economic needs of the colony's inhabitants. Additionally, the taste for own culture and segregation assumed in the concept of “parallel societies' has been criticized for neglecting immigrants” disadvantages on housing markets, but also for normative reasons (Secchi and Herath, 2019, p. 3). If immigrants' residential choices were driven by preferences for own-group neighbors, they would likely end up in homogenous ethnic minority neighborhoods. Given that in Germany ethnically homogeneous residential areas are not common (Schönwalder and Söhn, 2009), in contrast to spatial patterns in the U.S., they might have at least some “taste for diversity” (Dancygier and Laitin, 2014, p. 58). Whether they are interested in own-ethnic cultural and social capital or not, they tend to live in ethnically diverse neighborhoods.

The spatial clustering of ethnic minorities can emerge for various reasons—even without immigrants' preference for embeddedness into local own-group networks, e.g., due to stratified housing markets and discriminatory practices in the provision of housing (Lersch, 2013; Horr et al., 2018). Inequality in the access to housing markets becomes also obvious by the fact that many immigrants cannot realize their preferences with respect to proximity to urban green spaces (Kabisch and Haase, 2014). Furthermore, ethnic residential segregation might be a result of “*white flight*” processes (Crowder, 2000; Quillian, 2002; Goyette et al., 2014), which is a self-reinforcing outflow of better educated higher-status families from neighborhoods with a high concentration of ethnic minorities. This form of selective mobility might drain off resources and social capital from the local community and thereby increase neighborhood disadvantage (Sampson and Raudenbush, 2004). Increasing disadvantage leads to a decrease in housing prices, which cause a selective inflow of poorer people, who are often migrants.

The “*ethnic colony”* hypothesis assumes that immigrants are particularly interested in local ethnic or religious social capital, and therefore prefer to live with their co-ethnics. Alternatively, immigrants could have such a preference, but are unable (or unwilling) to realize their preferences by residential relocations if “competing” benefits outweigh the utility of ethnic local capital. For instance, immigrants might prefer to live close to co-ethnics and relatives, but also prefer neighborhoods with good primary schools for their children—which they would often find in other neighborhoods. Especially for long-term residents or 2nd or 3rd generation migrants, processes of structural assimilation might also lead to spatial assimilation and therefore cause a demand for better housing conditions (Häußermann and Siebel, 2000, p. 207; Lersch, 2013). Accordingly, the longer migrants stay in Germany, the more likely they will adapt their housing needs to the native population. Therefore, we assume that migrants' family formation changes the evaluation of the neighborhood to more child-related aspects, e.g., proximity to a desired primary school. Since we know from research that migrant parents have high educational aspirations for their children (Becker and Gresch, 2016), we expect that they, like native parents, also evaluate the spatial educational infrastructure when choosing a new dwelling.

Qualitative studies observe selective relocations of Turkish middle class families to neighborhoods with a lower share of ethnic minorities. For this group, moving to a new dwelling is motivated by the parents' desire to realize access to high-quality educational infrastructure. Concerns about the extent of school segregation and low achievement levels in adjacent schools motivate Turkish families to leave ethnic neighborhoods (Horr, 2008; Hanhoerster, 2015). Preferences for proximity to ethnic infrastructure, such as grocery stores or mosques, as well as to ethnic social networks, seem to be of secondary importance, especially for young parents (Horr, 2008, p. 190). Even if integration into the ethnic community and access to ethnic infrastructure were important factors for migrant families, this preference does not necessarily require physical proximity to ethnic neighborhoods in times of modern transportation and communication technologies (Zelinsky, 2001). “An ethnic grocer across town can easily be reached by bus once a week; friends or family members can be called every few days; and important community gatherings can be attended anywhere in the region on occasion” (Drever, 2004, p. 1,436).

Qualitative interviews conducted by Wiesemann (2008) with Turkish immigrants in Germany show that the ethnic character of a neighborhood plays an important role when choosing a location. However, ethnic preferences are in opposite directions: Whilst some households in his study preferred to live in areas with predominantly German natives, others chose to live in neighborhoods with large numbers of Turkish migrants, either because of the intra-ethnic contact opportunities or due to financial constraints. Taken together, these qualitative studies underscore that immigrants seem to have at least one important motive in common with natives, namely the preference for a “good” environment for their children, which is characterized by the absence of neighborhood disorder and the presence of high quality educational institutions. During family formation the evaluation of the neighborhood and the decision where to relocate might be similar compared with natives: in the end, it might be “moving for the kids,” rather than “moving for diversity.”

To test whether the local educational infrastructure is related to relocation behavior, we include the spatial proximity to a desired primary school in our analysis. Controlling for the evaluation of neighborhood characteristics, a strong effect of the absence of the desired primary school on residential moves would be an indicator of status attainment-motives. It is yet an empirical question whether patterns of residential relocations during family formation are either more in line with the “*ethnic colony”* hypothesis, or with spatial assimilation, motivated by better conditions for educational attainment for the children.

## Data and Methods

In our survey conducted in 2017 and 2018 in the federal states of Bremen, Lower Saxony, and North Rhine-Westphalia we asked mothers of children in 2nd and 3rd primary school grades about their residential biography, including their perceptions of neighborhood characteristics. Our window of observation begins with the date of moving into the dwelling where the female respondent became pregnant with the first child. Since respondents were required to recall neighborhood characteristics retrospectively, the survey instrument strongly benefitted from the idea of “cognitive anchors” (Loftus and Marburger, 1983). Even events that occurred rather distant in the past can be remembered if they relate to a significant other event, such as pregnancy or childbirth. To prevent distorted or erroneous memories, it is important that the interviewee has lived together with the child since birth, which is why we asked the biological mothers to complete the questionnaire. In 91.50% of cases this requirement was met, in 7.5% of cases the questionnaire was completed by the fathers, in 0.25 and 0.7% of cases by the stepmother/nursing mother and stepfather, respectively (Oeltjen and Windzio, 2019).

**Figure 1:**
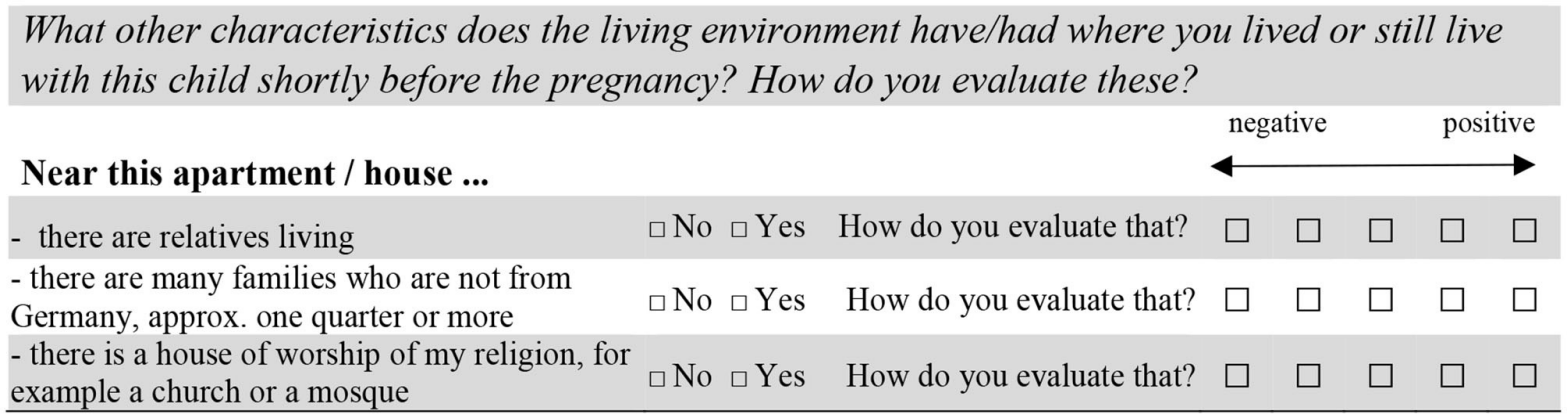
Survey instrument measuring three context characteristics and their evaluation.

The survey question for the first residential episode of interest was the following: “First of all, please think back to the time shortly before the pregnancy with the oldest child, i.e., with the 1st child in your household. Where did you live at that time? Please tell us the name of the town or city, the district or part of the town or city, and the time when you moved there.” For each dwelling the respondents were then asked to provide information on year and month they moved in, on characteristics of the living environment and to rate these characteristics on a five-point scale. Figure 1 gives an example of how we measured the presence of specific neighborhood characteristics and their evaluation. Regarding the neighborhood characteristics, we included the perception of relatives and migrant families living nearby, the perception of having a house of worship of the respective religion nearby as well as the proximity to a desired primary school. Since we assume that both the evaluation of neighborhood characteristics and the relocation rate (see below) might depend on perceived neighborhood disorder, we built a scale of neighborhood disorder by using factor analysis based on tetrachoric correlations among five binary items, which we show in Table A2 in the Appendix. The higher the value, the higher is the level of perceived disorder. Detailed descriptive statistics for the independent variables are shown in Table A3 in the Appendix.

Most studies lack information on the subjective assessment of neighborhood characteristics (Crowder, 2000; Schönwalder and Söhn, 2009; Lersch, 2013). Our procedure allows combining the (subjective) information of whether a characteristic existed in the respective neighborhood or not with the respondent's evaluation of this characteristic from her (or his) point of view. By doing so, we measure the subjective assessment of the neighborhood characteristics. For instance, respondents can live either with or without many immigrant neighbors and can evaluate the situation as it is. We rescaled the “positive” vs. “negative” continuum by centering each scale on its mean value. Mean-centering the scale does not change its interpretation: the higher the value, the more *positively* a respective characteristic is evaluated (Figure 1).

We distinguished two categories of migration background, namely “Turkish, Arabic and African” and “other migrants.” If a respondent reported that she was not born in Germany, or does not communicate with the child predominantly in German language, or if she completed the questionnaire in Turkish or Arabic language, we assume a respective migration background. We are well aware that our classification is very simple and that the category “other migrants” suggests a homogeneity, which is in reality inexistent. By considering also the language predominantly spoken at home, however, we capture an important indicator of ethnic background, which is usually ignored by categorizations applied in official statistics (Will, 2019)^1^. Moreover, residential mobility is a rare event. Since the computation of the hazard rate (see below) results from the number of events divided by the “time-at-risk” for each response-pattern in the explanatory variables, applying a more fine-grained distinction of ethnic groups is not possible.

For the analysis of the evaluation of neighborhood characteristics we use an ordered probit Heckman model (Greene and Hensher, 2010). The ordinal outcome of this model is the evaluation of neighborhood characteristics which we estimate for the selective subsample of those respondents who *have* the respective characteristic nearby in their neighborhood. In other words, the Heckman model takes into account the respondent's selection into a particular neighborhood. This selection process precedes the evaluation of neighborhood characteristics. If the selection into certain neighborhoods is not taken into account, the positive evaluation of a given state would indicate the appreciation of either the presence *or* absence of a particular characteristic, which is uninformative. The Heckman model accounts for the selection by weighting the effects of explanatory variables x_i_ on the positive evaluation by the process of selection into the respective state, which is influenced by the covariate vector **z**_i_. While the ordered probit model estimates the probability of a particular value *v*_*h*_ on the ordinal scale, which is the probability that **x**_*j*_*b*+*u*_1*j*_ falls between the cutpoints defined by θ (upper panel in Table 1), the term *s*_*j*_ in the binary probit selection equation (lower panel in Table 1) equals 1 when the respective characteristic exists in the respondent's neighborhood, and zero otherwise.

**Table 1:** Positive evaluation of neighborhood characteristics, ordinal probit model with Heckman selection.

	**(1)**	**(2)**	**(3)**	**(4)**
	**House of worship nearby**	**Many Non-Germans nearby**	**Relatives nearby**	**Desired school nearby**
**EVALUATION OF NEIGHBORHOOD (ORDINAL)**				
Migrant, Turk., Arab., Afric.	0.812^***^	0.498^***^	−0.199	−0.062
Migrant, other	0.288^*^	0.171^+^	0.036	0.022
Resp.: university degree	0.043	0.326^***^	−0.042	−0.110^*^
Educ. aspiration: university–entrance diploma	0.079	0.055	0.138^*^	0.163^**^
Dwelling: property	0.121^*^	−0.115^+^	−0.125	−0.102^+^
**SELECTION INTO NEIGHBORHOOD (BINARY)**				
Bremen	−0.053	0.249^**^	−0.152^+^	0.184^**^
NRW	0.205^***^	0.121^*^	0.232^***^	0.111^**^
Resp.: male	0.169	0.115	0.044	0.087
Age at family formation	0.008	−0.007	−0.037^***^	0.004
Unemployment in household	−0.023	0.185^**^	−0.006	0.031
Migrant, Turk., Arab., Afric.	−0.181^*^	0.278^**^	0.085	0.254^**^
Migrant, other	−0.370^***^	0.203^**^	−0.210^**^	0.016
Resp.: university degree	0.051	0.140^*^	−0.415^***^	−0.084^+^
Educ. aspiration: university–entrance diploma	−0.085^+^	0.029	−0.113^*^	−0.009
Dwelling: property	0.225^***^	0.008	0.493^***^	0.516^***^
Dwelling: close to workplace	0.187^***^	−0.039	0.096^*^	0.257^***^
Perceived disorder	0.388	4.748^***^	−1.091^+^	0.927^+^
Perceived disorder^2^	−0.033	−0.889^***^	0.280	−0.463^*^
Constant	−0.487	−5.257^***^	1.994^***^	−0.843^*^
Cut1	−1.915^***^	−0.985^***^	−2.305^***^	−2.801^***^
Cut2	−1.465^***^	−0.397^***^	−2.083^***^	−2.629^***^
Cut3	0.329^+^	1.080^***^	−1.366^***^	−1.667^***^
Cut4	0.934^***^	1.649^***^	−0.622^*^	−0.947^***^
Athan rho	0.278	0.222^***^	−0.190	−0.820^***^
Observations	8,612	8,785	8,856	8,623

+ *p < 0.1*,

* *p < 0.05*,

** *p < 0.01*,

*** *p < 0.001, corrected standard errors for clustering in respondents*.

Source: DFG–Project “Moving for the Kids,” own calculations.

$$\begin{array}{l} \text{Pr}\left(y_{j}=v_{h}\right)=\text{Pr}\left(\theta_{k-1}<{\text{x}}_{j}b+u_{1j}<\theta_{k}\right)\;s_{j}=1\left({\text{z}}_{j}\gamma +u_{2j}>0\right) \end{array}$$

The two error terms (*u*_1*j*_, *u*_2*j*_) are assumed to have a bivariate normal distribution with zero mean, a variance of 1 and a covariance of ρ.

$$\begin{array}{l} \left[\begin{array}{cc} 1 & \rho \\ \rho & 1 \end{array}\right] \end{array}$$

If ρ equals zero, standard ordered probit models will provide unbiased results, but if ρ≠*0*, the model corrects for selection bias. Unlike the classic Heckman selection model, however, ordered probit Heckman models do not compute a λ term. In the classic Heckman model, λ is an estimate of the selection process by computing the Inverse Mills ratio of **z**_*j*_γ, which is a hazard rate-like representation of non-selection into the observed sample. In contrast, the ordered probit Heckman model takes unobserved heterogeneity shared by both equations into account by controlling for the correlation of the error terms (Greene and Hensher, 2010, p. 308). The model is thus similar to the bi- or multivariate probit models. The strength of this correlation indicates the magnitude of the component of unobserved heterogeneity that is shared by both equations, namely the selection and the evaluation.

Having analyzed the evaluation of neighborhood characteristics conditional on the selection process, we investigate in a subsequent step the effects of these evaluations on residential relocations. Residential relocations are events occurring after some waiting-time, beginning with moving into a dwelling and ending with the event of moving out. Since the information on the residential biography has been collected on a monthly basis, the resulting time-to-event data allows to predict the transition from the initial state “not moved” into the destination state “moved”. We thus apply event history analysis. Here, the hazard rate *r(t)* is the outcome of interest, which is the (conditional and time-specific) relation of the number of events *f(t)* in the nominator and time at risk (months) of those who have not yet experienced the event of relocation *G(t)* in the denominator, formally *r(t)* = *f(t)/G(t)*. At each point in time *t*, right censored observations without an event contribute to the denominator of the (time-dependent) ratio of events to risk time (Windzio, 2013, p. 124). Since the event of interest is a residential relocation during the period of family formation, we will refer to “relocation rates.” These relocation rates are the outcomes in the event history regression models. However, we are also interested in relocations triggered by particular motives, namely by improving the social context or for family reasons. Accounting for different motives of relocations is important in our study since we are interested in particular preferences of migrants and natives. We regard these different motives as competing risks. Accordingly, a relocation can be motivated either by improving the social context or by family reasons, and these two outcome-events “compete” against each other for occurrence. We defined relocations motivated by the “social context” by a set of items where respondents reported the reasons for a relocation. In order to identify the “improvement of social context” as a motive, we combined the following statements: the respondent wished to live “in a better social environment,” “with lower cultural diversity” and “nearby the desired school.” In addition, we enhanced this measurement with information from an open-ended category where respondents reported their motives in their own words, e.g., saying that they lived in an “unsafe,” “bad” or “noisy” neighborhood or with many “non-German citizens.” We defined the destination state “relocation, family” by reasons related to marriage and divorce and added information from open-ended questions on e.g., “parents,” “relatives,” and “family.” In a competing risk analyses we get different coefficient vectors for each competing risk (see **Table 3**).

Our observations are clustered in residential areas, namely in 585 different localities, that is, towns, cities, and villages. In order to account for the statistical non-independence of observations in these localities, we apply multilevel Weibull models of event history analysis, which enhances the standard Weibull model with a random intercept *u*_*j*_. The term *u*_*j*_ is constant within the contexts, and varies between contexts.

$$\begin{array}{l} r\left(t_{ij}\right)=r_{0}\left(t_{ij}\right)•\text{exp}\left({\text{x}}_{ij}\beta +u_{j}\right) \end{array}$$

In the Weibull model (here in proportional hazards notation), the hazard rate *r*(*t*_ij_) estimates hazard ratios relative to a baseline hazard *r*_0_(*t*_ij_). These hazard ratios might depend on unobserved characteristics of residential places (cities or villages), captured by the random effect *u*_j_.

In the Heckman model and in the event history model we do not control for income, but for high education, unemployment and home ownership. It is hard to get reliable information on income in a self-administered survey. Moreover, respondents' cognitions and subjective perceptions correlate with education (Loftus and Marburger, 1983) rather than with income, which is why we do not necessarily need the income variable. We also include the squared value of perceived disorder into our models in order to allow for non-linear affects, e.g., declining effects at higher values of disorder.

## Results

In the first part of our empirical study we test whether immigrants and natives evaluate particular neighborhood characteristics differently. Subsequently, we analyse the effects of these evaluations on residential relocations. Table 1 shows the two components of the Heckman model: the effects of the ordered probit model on the evaluation of neighborhood characteristics (upper panel) and the effects of the binary probit selection model (lower panel). The upper part of the model does not control for many confounders because we assume that economic factors (e.g., unemployment) do more account for the location in a respective neighborhood, rather than for the cognitive process of evaluating its characteristics (see section Data and Methods).

Results show that migrants of Turkish, Arabic or African origin tend to evaluate proximity to a *house of worship* of their religion more positively than the reference group of non-migrants. The same is true for the category “Migrant, other,” albeit the effect is smaller in magnitude. We find a similar pattern in the evaluation of *living with many Non-Germans nearby*. While the effect of “Turkish, Arabic or African origin” is significant and positive, it is significant only at the 10% level for other migrants. Regarding the evaluation of living with *relatives nearby*, we do not find any difference between non-immigrants and immigrant groups: on average, proximity to relatives is evaluated similarly in all three groups. High educational aspiration, i.e., the expectation that the child will graduate from high school with the *Abitur*^2^, increases the positive evaluation of *relatives nearby* and *desired school nearby*. Since our sample is biased with respect to educational attainment (Oeltjen and Windzio, 2019), we cannot rule out that this effect also results from dual-earner families with high educational aspirations, where employed parents appreciate the proximity to e.g., their children's grandparents who regularly care for their children. Overall, the basic pattern of covariate effects on positively evaluating the *desired school nearby* is more or less similar to the pattern found for *relatives nearby*: again, there is no significant difference between non-immigrants and our two immigrant groups.

Furthermore, while families living in residential property tend to appreciate a house of worship nearby, they tend to deprecate living with many Non-Germans in the neighborhood, but the latter effect is significant at the 10% level only. Similarly, living in residential property reduces the positive evaluation of having the desired school nearby. Although the effect is significant at the 10% level only, it is rather counterintuitive, since property has a robust positive effect of selection into such neighborhoods.

In the selection part of the model, results show that respondents in both immigrant categories tend to live significantly more often in neighborhoods where they perceive many Non-Germans nearby, so these effects reflect micro-level manifestations of immigrant residential segregation. The same is true for the positive effect of unemployment in the household on living with many Non-Germans, which is in line with findings showing strong correlation of high shares of immigrants and socio-economic deprivation at the aggregate level of neighborhoods (Teltemann et al., 2015). This interpretation corresponds well with the very strong effect of perceived neighborhood disorder on the propensity to live with many Non-Germans nearby.

Due to the simultaneous inclusion of its squared value *(perceived disorder*^2^), the effect is positive in particular at lower values of disorder, but dampens at higher values. Interestingly, there is a significant positive effect of respondents' higher education (university degree) on living with *many Non-Germans nearby*, which possibly results from the fact that higher educated respondents tend more to live in urban areas, where the exposure to ethnic and cultural diversity in their neighborhoods is higher. Additionally, it is more unlikely for respondents with university degree to live nearby relatives. This is not surprising, since academics tend more to long-distance relocations for job reasons, which is often accompanied with a higher spatial distance to other family members.

**Figure 2:**
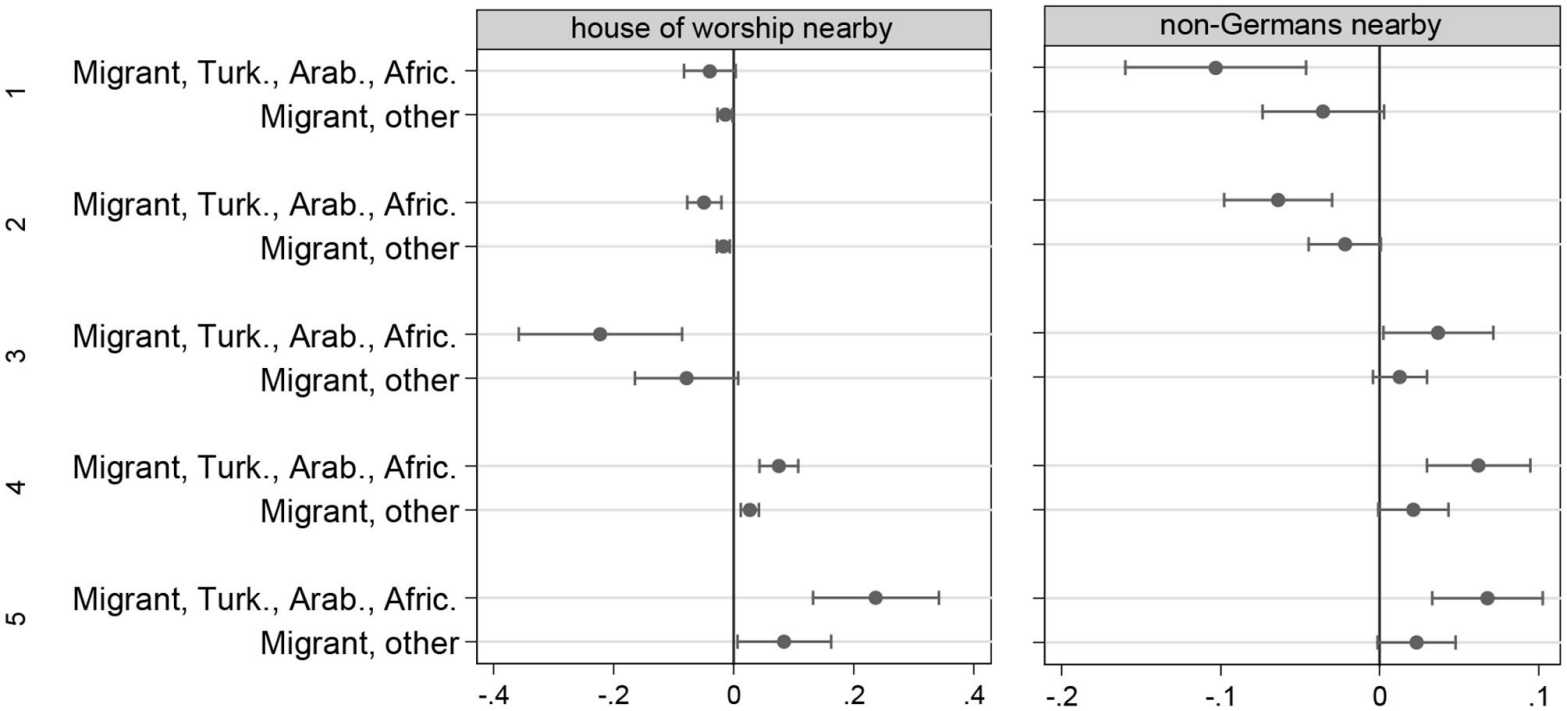
Average marginal effects of the immigrant categories, results from Table 1.

Figure 2 shows average marginal effects (AMEs) of the immigrant categories on the positive evaluation of *living with many Non-Germans nearby* and on living nearby a *house of worship*. The vertical line represents the non-immigrant reference group (=0). For each category of the ordinal outcome variable, the error bar represents the AME of the respective immigrant category. Regarding living nearby a *house of worship*, migrants of Turkish, Arabic and African origin show a significantly reduced probability of categories 2 (−0.049^**^) and 3 (−0.222^**^) of the dependent variable, while the probabilities of categories 4 (0.075^***^) and 5 (0.236^***^) are significantly increased: they systematically tend to more positive evaluations of *having a house of worship nearby*. Similarly, the probability of category 1 for the evaluation of *living with many Non-Germans* nearby is reduced for migrants of Turkish, Arabic and African origin (-0.103^***^), also the probability of category 2 (-0.063^***^), whereas the probabilities of categories three and higher are increased (0.036^*^; 0,062^***^; 0.067^***^). Regarding the group of “other migrants,” we also find a tendency toward a more positive evaluation of many Non-Germans in the neighborhood, but the effects differ less strongly from the reference group of native persons (the center line). Overall, first and later immigrant generations of Turkish, Arabic or African origin seems to have a positive attitude toward ethnic-religious cultural and social capital (Esser, 2010), and a preference for diversity rather than to spatial assimilation (Massey and Denton, 1985).

To sum up, we do find differences in the evaluation of neighborhood characteristics between immigrants and natives. However, these differences are limited to the evaluation of having a house of worship nearby and living with many Non-Germans nearby, whereas we do not find systematic group differences between the evaluation of living with relatives nearby and having the desired school nearby.

In the next step, we are interested in whether these differences in neighborhood evaluation influence residential relocations. In Table 2, the multilevel event history Model (1) shows the overall patterns, whereas the second Model (2) applies interaction terms to disentangle the effects of neighborhood evaluation between natives and immigrants. Both models show negative effects of *age at family formation* on the relocation rate (hazard ratio <1). In line with common expectations, families who live in their residential property are much less mobile than families in the reference group (tenants).

**Table 2:** Effects of neighborhood evaluation on relocation rates, multilevel Weibull models, hazard ratios.

	**(1)**	**(2)**
	**Relocation**	**Relocation**
*n* previous episodes	1.575^***^	1.569^***^
Bremen	1.283^*^	1.271^*^
NRW	0.929	0.930
Resp.: male	0.986	0.978
Age at family formation	0.981^***^	0.981^***^
Unemployment in household	1.106^+^	1.112^+^
Migrant, other	0.841^**^	0.879^+^
Migrant, Turk., Arab., Afric.	0.689^***^	0.597^***^
Resp.: university degree	1.282^***^	1.280^***^
Educ. aspiration: university-entrance diploma	1.125^**^	1.129^**^
Dwelling: property	0.254^***^	0.254^***^
Dwelling: close to workplace	0.979	0.977
6 months ± new child	2.662^***^	2.660^***^
3 months ± new job	1.528^***^	1.526^***^
Desired school *not* in neighborh.	1.812^***^	1.816^***^
Perc. neighborh. disorder	1.010	1.010
**POSITIVE EVALUATION OF NEIGHBORH. CHARACTERISTICS**		
Eval. house of worship	0.960	0.966
Eval. many Non-Germans	1.010	0.987
Eval. relatives	0.908^***^	0.896^***^
**INTERACTION TERMS**		
Eval. house of worship X Turk., Arab., Afric.	−−	1.055
Eval. many Non-Germans X Turk., Arab., Afric.	−−	0.952
Eval. relatives X Turk., Arab., Afric.	−−	1.277^*^
Eval. house of worship X oth. migrant	−−	0.915
Eval. many Non-Germans X oth. migrant	−−	1.176^+^
Eval. relatives X oth. migrant	−−	0.972
Constant	0.003^***^	0.003^***^
log(rho)	0.123^***^	0.123^***^
var(level 2: city)	0.037^*^	0.034^*^
*N* events	2,422	2,422
Observations	23,375	23,375

Exponentiated coefficients

+ *p < 0.1*,

* *p < 0.05*,

** *p < 0.01*,

*** p < 0.001.

Source: DFG-Project “Moving for the Kids,” own calculations.

In addition, both models indicate that respondents of our two immigrant categories tend to lower relocation rates. In contrast, respondents with higher education (university degree) and higher educational aspiration for their children are more inclined to relocate during family formation. Unsurprisingly, the effects of time-varying covariates “±6 months before and after giving birth of a subsequent child” and “± 3 months before and after starting a new job” affect relocation rates positive and are significant. In addition, the absence of the desired school in the neighborhood considerably increases the relocation rate. This finding provides clear evidence that the local educational infrastructure has an effect on relocation decisions.

Both models include three effects of neighborhood evaluation: having a house of worship nearby, living with many Non-Germans, and proximity to relatives. In Model (1) the main effect of a positive evaluation of proximity to relatives has a significantly negative effect on relocations, whereas the other two effects are insignificant. We find an interaction effect in Model (2) in opposite direction to the negative main effect (of the positive evaluation of relatives nearby) for immigrants of Turkish, Arabic and African origin. This is in contrast to our expectation: if this particular group were more interested in living close to their own ethnic relatives than natives are, this “bonding social capital” (Putnam, 2000) should have resulted in a negative interaction effect, so that these immigrants would have been even *more* immobile than natives when they appreciate proximity to relatives. Similarly, the interaction “eval. many Non-Germans X oth. Migrants” is positive, but significant just at the 10% level. In both cases it seems that the interaction effects result from relocations which are not in line with the assumption that immigrants were particularly interested in “bonding social capital” to their ethnic group or their family (Putnam, 2000).

Models in Table 3 estimate effects of neighborhood evaluation on relocation rates in a competing risk perspective. Models (1) and (2) show effects on relocations aiming at *improving the social context*, Models (3) and (4) estimate determinants of relocations for *family reasons*. Overall, respondents of both immigrant categories seem to be less mobile, which corroborates results from Model 1 in Table 2, but the hazard ratio is significantly <1 only for other migrants aiming at improving the social context in Model (1) (0.760^*^). Similarly, a higher educational aspiration for the children increases the rate of relocations for improving the *social context* (1.192^*^), but not for *family reasons*, whereas the intervening events of giving birth to a new child and changing the job tend to positive effects in all four models. This also holds for perceived neighborhood disorder, even though the effects seem to be stronger and more robust for *improving the social context* than for *family reasons*. A similar pattern results for the absence of a desired primary school: in all four models we find a significant and positive effect on the relocation rate, whereby this influence is clearly stronger for relocations aiming at improving the social context.

**Table 3:** Effects of neighborhood evaluation on relocation rates, multilevel Weibull models, hazard ratios, by reason of relocation.

	**(1)**	**(2)**	**(3)**	**(4)**
	**Relocation, social context**	**Relocation, social context**	**Relocation, family**	**Relocation, family**
Bremen	1.054	1.043	1.415^+^	1.423^+^
NRW	0.964	0.964	0.987	0.986
Resp.: male	0.934	0.915	0.856	0.854
Age at family formation	0.991	0.993	0.980^*^	0.981^*^
Unemployment in household	1.248^*^	1.283^*^	1.389^**^	1.406^**^
Migrant, other	0.760^*^	0.855	0.843	0.935
Migrant, Turk., Arab., Afric.	0.896	0.729	0.809	0.748
Resp.: university degree	0.886	0.895	1.127	1.120
Educ. aspiration: university-entrance diploma	1.192^*^	1.197^*^	1.072	1.076
Dwelling: property	0.396^***^	0.393^***^	0.373^***^	0.372^***^
Dwelling: close to workplace	0.838^*^	0.832^*^	1.062	1.054
6 months ± new child	3.471^***^	3.461^***^	4.478^***^	4.486^***^
3 months ± new job	1.414^+^	1.419^+^	1.815^***^	1.816^***^
Desired school *not* in neighborh.	2.969^***^	2.974^***^	1.910^***^	1.925^***^
Perc. neighborh. disorder	2.139^***^	2.098^***^	1.229^+^	1.241^+^
**POSITIVE EVALUATION OF NEIGHBORH. CHARACTERISTICS**				
Eval. house of worship positive	0.937	0.930	0.941	0.994
Eval. many Non-Germans positive	0.878^*^	0.795^***^	1.034	1.010
Eval. relatives positive	0.895^*^	0.870^*^	0.867^**^	0.858^**^
**INTERACTION TERMS**				
Eval. house of worship X Turk., Arab., Afric.	−−	1.316	−−	0.933
Eval. many Non-Germans X Turk., Arab., Afric.	−−	1.119	−−	0.895
Eval. relatives X Turk., Arab., Afric.	−−	1.273	−−	1.214
Eval. house of worship X oth. migrant	−−	0.815	−−	0.656^*^
Eval. many Non-Germans X oth. migrant	−−	1.714^***^	−−	1.372^+^
Eval. relatives X oth. migrant	−−	1.084	−−	0.973
Constant	0.001^***^	0.001^***^	0.002^***^	0.002^***^
log(rho)	0.055+	0.058+	0.003	0.003
var(level 2: city)	0.029	0.033	0.082^**^	0.078^**^
*N* events	672	672	700	700
Observations	17,056	17,056	17,056	17,056

Exponentiated coefficients

+ *p < 0.1*,

* *p < 0.05*,

** *p < 0.01*,

*** p < 0.001.

Source: DFG-Project “Moving for the Kids,” own calculations.

Again, living in residential property has a consistently negative effect on all competing risks, whereas proximity to the workplace has a negative effect only on relocations motivated by relocations for improvement of the *social context*. Interestingly, the positive evaluation of many Non-Germans in the neighborhood points in the opposite direction for other migrants. While the effect is negative for non-immigrants (0.795), it is even positive for other migrants (0.795^*^1.714 = 1.362). In other words, even though other migrants appreciate the presence of many Non-Germans in the neighborhood, they show an increased tendency to relocate in order to change their neighborhood context. We find a similar pattern for relocations for family reasons, but the positive effect of “eval. many Non-Germans X oth. migrants” is only significant at the 10% level. At the same time, the insignificant main effect is close to 1, which means, there is no effect on the transition into the state “moved”.

The relocation rate decreases in all four models the more positive respondents evaluate the proximity to relatives. If they appreciate the presence of many Non-Germans, the relocation rate decreases when motivated by *improvement of the social context*, whereas the effect is insignificant with respect to relocations for *family reasons*. Accordingly, even though we found differences between immigrants and natives in the *evaluation of neighborhood characteristics*, we do *not* find corresponding *relocation patterns*. Although evaluations of neighborhood characteristics play a role for the decision to relocate, e.g., by a consistently negative effect of appreciating that relatives live nearby, effects of these evaluations on actual residential moves do not differ between immigrants and natives^3^.

**Figure 3:**
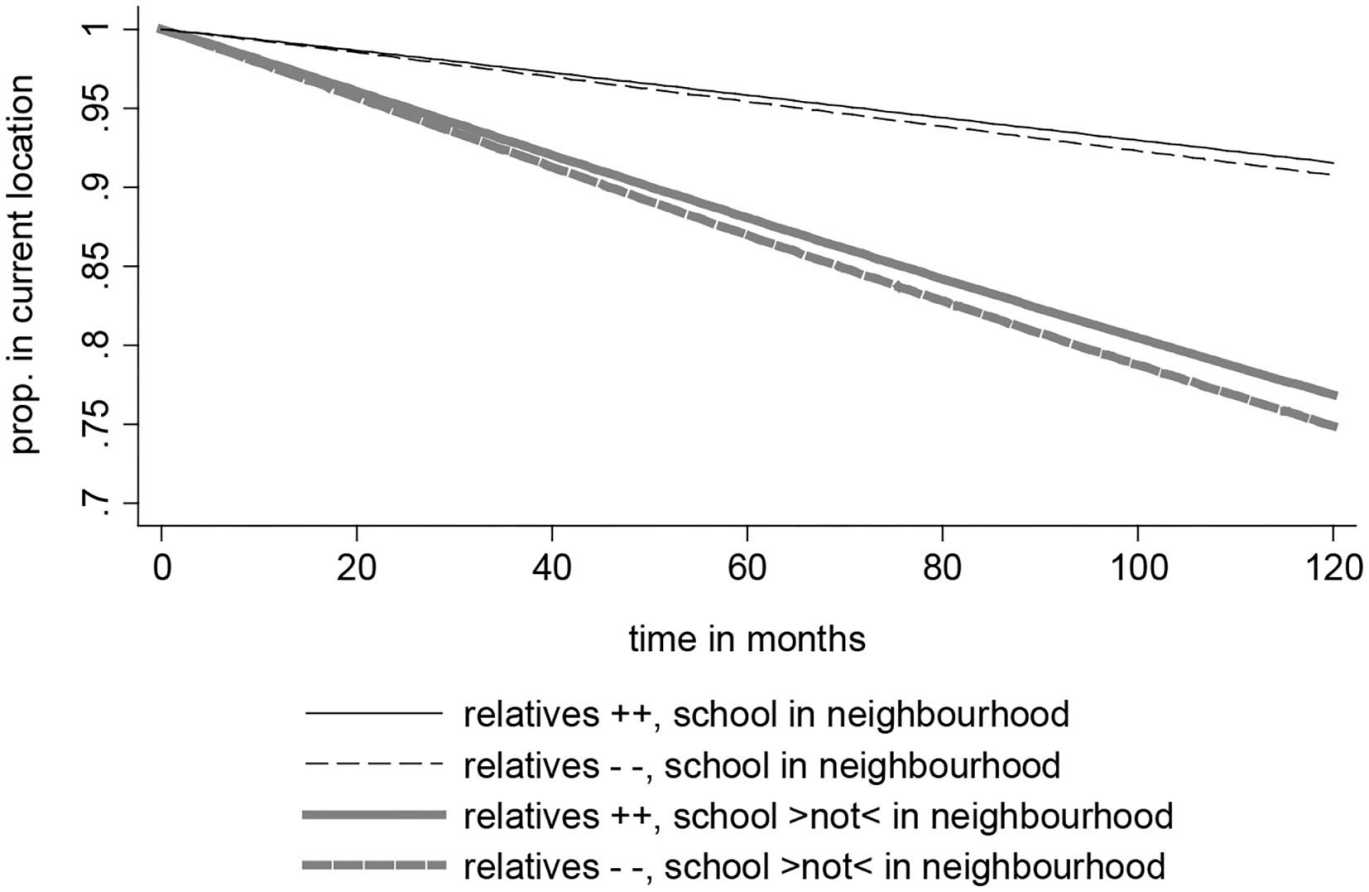
Relative effect strength of “desired school not in neighborhood” vs. “positive evaluation of relatives living nearby” on relocations for improvement of social context, survivor functions.

Moreover, being aware of the strong effect of the absence of a desired primary school, respondents might evaluate their neighborhood during family formation primarily with respect to the socialization of their children. It is thus interesting to compare the strength of these two effects on relocations motivated by improving the social context: first, the effect of appreciating relatives nearby, secondly, the effect of not having the desired school in the neighborhood. Which of these effects is stronger and thus more relevant (Figure 3)?

Figure 3 shows post-estimation results from the single level Weibull model (Table A1, Appendix) of relocations aiming to improve the social context. Instead of focusing on significance, predicted survivor functions provide a clear insight into the relevance of the effects on interest. In Figure 3, the survivor functions *G(t)* indicate for each month the share of episodes without an event of relocation (“in current location”). For the prediction we held all control variables constant at their mean values. Technically, the prediction results from the antilog of the negative accumulation of the hazard rate over time (Windzio, 2013, p. 120).

$$\begin{array}{l} \text{G}(t)=\text{exp}\left(-\int_{0}^{\text{t}}\text{r}(\tau )\text{d}\tau \right) \end{array}$$

In the predicted scenario the evaluation of relatives living nearby is either positive (++, mean evaluation +0.5 standard deviation) or negative (− −, mean evaluation−0.5 standard deviation). The visualization in Figure 3 highlights that the effect of not having the desired school in the neighborhood is much stronger than the positive evaluation of living with relatives nearby (comparison between the small and the thick lines). The thin black lines represent the situation when the desired school is nearby, the bold gray lines a scenario where the desired school is absent.

When the desired school is nearby, after 120 months 91.5% still live in the current location when they appreciate the presence of relatives nearby, and 90.7% who do not appreciate relatives nearby. When the school is *not* nearby, in contrast, the overall share of stayers is considerably lower: 76.8% of those who appreciate the presence of relatives nearby still live in the neighborhood after 120 months, and 74.8% of those who do not appreciate relatives in their neighborhood. Accordingly, the effect of the desired school is *very* strong, whereas the effect of social capital provided by family networks on relocations is comparatively small.

In summary, our results show that immigrants, particularly those of Turkish, Arabic or African origin, show a more positive evaluation of living nearby a house of worship of their religion and of living with many Non-Germans. Thus, regarding the evaluations there seems to be a preference for high diversity. However, during family formation these preferences do not transform into relocations conducted to realize these preferences: we found that preferences do indeed have an effect on actual rates of relocation, but the effects of these preferences do not differ between immigrants and natives in the way assumed according to the “ethnic colony” hypothesis. Following this hypothesis, a strong preference for ethnic or religious capital, such as houses of worship or ethnic and cultural diversity in the neighborhood, should decrease rates of residential relocations in particular for immigrants—which is empirically not the case. Surely, our results should be interpreted against the background of a considerable sampling bias toward respondents with higher education (Oeltjen and Windzio, 2019). Moreover, if residential segregation in combination with increasingly unequal housing markets were very strong, meaning that in general, relocations of immigrants across different types of neighborhoods rarely occur and immigrants mostly stay in highly diverse and often deprived neighborhoods before and after family formation, “ethnic colonies” would exist independently of the relocation dynamics we observe in our data.

## Summary and Conclusion

Many studies describe patterns and trends of segregation at the aggregate level, although Th. Schelling's macro-micro-macro explanatory mechanism is based on individuals' or households' behavior. In our study, we followed Schelling's analytic shift toward the micro-level. First, we analyzed respondents' evaluations of particular neighborhood characteristics. Second, we analyzed the potential effects of these evaluations on actual residential relocations. Following to the classic work of P. H. Rossi, who identified family formation and family enhancement as crucial drivers of residential relocations between different neighborhoods (Rossi, 1954), we were interested to find out whether different residential preferences in the phase of family formation and extension account for different relocation decisions between native and migrant families.

In the theoretical part of our study, we discussed the emergence of “ethnic colonies” or “parallel societies,” which assume that migrants had a preference for living close to other members of their ethnic community. Contrary to this theoretical argument our results show that during family formation immigrants' residential relocations do not indicate that “bonding social capital” within the own ethnic community or other immigrant groups is a basic driver of these relocations. On the one hand, immigrants and natives tend to evaluate characteristics of their neighborhoods differently, as we have shown in the first part of our empirical analysis. We found that particularly respondents of Turkish, Arabic or African origin highly appreciate living nearby a house of worship and also living with many Non-Germans. On the other hand, our analysis of how these evaluations transform into residential relocations did not show any differences between immigrants and natives. Evaluations or preferences during family formation do not trigger relocations that result in “ethnic colonies” at the macro level. First and foremost, both migrants *and* non-migrants seem to be sensitive to the educational infrastructure in their neighborhood. Aside from preferences toward ethnic “bonding social capital,” there are competing factors, for instance, whether the desired school exists in the neighborhood or not. From the immigrants' or the ethnic minorities' perspective, the issue of appreciating the educational infrastructure is related to investments into educational attainment and, into the process of intergenerational integration into the host society (Esser, 2010). As we could show by comparing effect sizes, absence of the desired school has a much stronger effect on relocations than the positive evaluation of proximity to relatives—a result which is indifferent toward immigrant origin. Relocations during family formation result from the same pattern of covariates in all three groups. According to an earlier study (Oeltjen and Windzio, 2019), residential segregation between immigrants and natives is also an outcome of different *destinations* where households relocate, that is, immigrants *and* natives are sensitive to neighborhood disorder and the absence of the desired school in the neighborhood and relocate, but immigrants end up again in neighborhoods where the situation is rather similar to the previous one.

Although we achieve robust and clear effects, we should also address the limitations of our study, which primarily result from the field access. Even though great importance was attached to the simplicity and clarity of the survey instrument, written surveys are particularly susceptible to measurement error due to the uncontrollability of the survey situation. In addition, retrospective information is not free from measurement error even if our instrument applies cognitive anchors.

Furthermore, despite knowing the individual place of residence, we didn't include any objective characteristics of the city or village, for example the population size. Even if we assume that subjective perceptions of the neighborhood are predominantly relevant for relocation decisions, we should keep in mind that these subjective perceptions are related to objective residential attributes. For example, the perception of neighborhood diversity or disorder is probably higher in urban areas compared with rural areas. In order to gain a better understanding of how subjective perceptions differ by regional contexts, objective information about the residential spaces should be included in the analysis.

To sum up, the most important result of our study is that immigrants seem to evaluate neighborhood characteristics related to “ethnic colonies” (living with relatives and with many Non-Germans nearby) differently from non-migrants, but they do not systematically translate these evaluations into specific relocation patterns. Educational infrastructure and proximity to relatives is important for migrants *and* non-migrants. Results also show that the absence of the desired school nearby has a much stronger effect on relocations than the preference of living close to relatives.

Even though our micro-level analyses show clear differences between immigrants and natives in the evaluation of neighborhood characteristics, and also explain the overall process of relocation, they do not systematically explain patterns of residential segregation. Our results indicate that relocations during family formation do not entail “ethnic colonies” at the aggregate level. Nevertheless, there are considerable degrees of segregation at the macro-level, which is not just a result of socio-economic inequality between immigrants and natives (Teltemann et al., 2015). In addition, while the causes of moving out of a particular neighborhood do not overwhelmingly vary between immigrants and non-immigrants, recent results show that the quality of the *destination* seems to differ, whereby this quality is measured by indicators of neighborhood disorder (Oeltjen and Windzio, 2019).

Future research should investigate in detail the micro-mechanisms of residential segregation in Germany, including the migrant and the native perspective, especially since residential segregation is related to processes of social integration. Same ethnic preferences and ethnic homophily with respect to social support and friendship choice (Windzio, 2018) are indicators of ethnic boundaries (Wimmer, 2013). If these boundaries contribute to the reproduction of group differences over time, they will also reproduce group-differences in language, norms, taken-for-granted-knowledge, and culture in general. Presumably, cultural differences between groups will correspond with differences in status attainment if culture is utilized as “capital” (Bourdieu, 1986). If cultural capital matters for social mobility it will be rather unlikely that cultural diversity is unrelated to unequal chances of status attainment. In the end, differences in cultural capital can result in intergroup conflicts (Windzio, 2016). In this regard, understanding ethnic residential segregation and segregation of social networks, as potential promoters for ethnic boundaries, will be important topics for future research.

## Data Availability Statement

The datasets generated for this study will not be made publicly available, due to data privacy legislation. The data collection involves schools and has been approved by the federal school authorities, conditional that the data is not accessible to 3rd persons. However, the data can be reanalyzed in our institute.

## Ethics Statement

Ethical review and approval was not required for the study on human participants in accordance with the local legislation and institutional requirements. The patients/participants provided their written informed consent to participate in this study.

## Author Contributions

MO did the field work, contributed major parts to the manuscript, and joint the development/discussion of the research question. AB contributed to the manuscript. MW contributed major parts to the manuscript, joint the development/discussion of the research question, and did the computation. All authors contributed to the article and approved the submitted version.

## Conflict of Interest

The authors declare that the research was conducted in the absence of any commercial or financial relationships that could be construed as a potential conflict of interest.

## Footnotes

^1^Additionally, we assume that for migrants and native families the same categories are relevant for neighborhood evaluations, regardless of whether they are rated positive or negative. Theoretically, it is possible that, the subjective neighborhood evaluation of migrants and native persons is formed by different categories and their respective perception. Since we had to define the categories for which we asked for an evaluation, we were not able to consider potential differences in evaluation categories.

^2^The Abitur is the highest school leaving examination in Germany and qualifies for university entrance.

^3^If we enhance Model A1, Appendix, with the evaluation of having the desired school nearby and interaction terms with both immigrant categories (model not shown), we do not find any significant or notable difference between immigrants and natives.
