# Supplementary material for: Moving for Diversity or Moving for the Kids? The Micro-Dynamics of Residential Relocations During Family Formation of Immigrants and Natives
Source: Front Sociol. 2020 Oct 26;5:538946. doi: 10.3389/fsoc.2020.538946 (PMC8022484; doi:10.3389/fsoc.2020.538946)
Supplement: Supplementary file 1 [file Data_Sheet_2.docx]

# Appendix

Table A1: Effects of neighbourhood evaluation on relocation rates, Weibull model, hazard ratios, relocation for improvement of social context

|  | (1) |
| --- | --- |
|  | relocation, social context |
|  |  |
| Bremen | 1.037 |
| NRW | 0.959 |
| resp.: male | 0.941 |
| age at family formation | 0.992 |
| unemployment in household | 1.271* |
|  |  |
| Migrant, other | 0.760* |
| Migrant, Turk., Arab., Afric. | 0.888 |
|  |  |
| resp.: university degree | 0.883 |
| educ. aspiration: university-entrance diploma | 1.194* |
| dwelling: property | 0.394*** |
| dwelling: close to workplace | 0.837* |
| 6 months +/-new child | 3.488*** |
| 3 months +/-new job | 1.411+ |
|  |  |
| desired school *not* in neighbourh. | 2.986*** |
| perc. neighbourh. disorder | 2.129*** |
|  |  |
| ***positive evaluation of neighbor. characteristics*** |  |
| eval. house of worship positive | 0.947 |
| eval. many non-Germans positive | 0.882* |
| eval. relatives positive | 0.891* |
| Constant | 0.001*** |
| log(rho) | .050 |
| N events | 678 |
| Observations | 17286 |

Exponentiated coefficients + *p* < .1, * *p* < .05, ** *p* < .01, *** *p* < .001

Source: DFG-Project „Moving for the Kids“, own calculations

Table A2: Items in factor analysis of perceived disorder in the neighbourhood based on polychoric correlations N= 7202 episodes, Cronbach's alpha= 0.67

| Item  Near this apartment/house… | Factor loadings |
| --- | --- |
| … are many families who barely speak German  ... is dirt and trash on the sidewalks  … is a loud atmosphere outside  … are many families from different cultures  … are few rather poor families (r) | .83  .80  .78  .81  -.40 |

Source: DFG-Project „Moving for the Kids“, own calculations

Table A3: descriptives Statistics

| variable | N | mean | sd | min | max |
| --- | --- | --- | --- | --- | --- |
| evaluation: house of worship nearby | 8293 | 3.5 | 0.95 | 1 | 5 |
| evaluation: many non-Germans nearby | 8336 | 3.36 | 0.92 | 1 | 5 |
| evaluation: relatives nearby | 8361 | 3.47 | 1.51 | 1 | 5 |
| evaluation: desired school nearby | 7995 | 3.65 | 1.35 | 1 | 5 |
| age at family formation * | 8521 | 29.12 | 5.3 | 12 | 50 |
| resp.: male | 8521 | 0.07 | 0.25 | 0 | 1 |
| resp.: university degree | 8521 | 0.39 | 0.49 | 0 | 1 |
| resp.: migrant | 8454 | 0.2 | 0.4 | 0 | 1 |
| Migrant, Turk., Arab., Afric. | 8521 | 0.06 | 0.24 | 0 | 1 |
| Migrant, other | 8521 | 0.14 | 0.35 | 0 | 1 |
| educ. aspiration: university-entrance diploma | 8521 | 0.55 | 0.5 | 0 | 1 |
| 6 months +/-new child | 8521 | 0.22 | 0.42 | 0 | 1 |
| 3 months +/-new job | 8521 | 0.21 | 0.41 | 0 | 1 |
| dwelling: property | 8521 | 0.38 | 0.49 | 0 | 1 |
| Bremen | 8521 | 0.09 | 0.29 | 0 | 1 |
| NRW | 8521 | 0.45 | 0.5 | 0 | 1 |
| desired school *not* in neighbourhood | 8300 | 0.48 | 0.5 | 0 | 1 |
| dwelling: close to workplace | 8521 | 0.43 | 0.5 | 0 | 1 |
| unemployment in household | 8521 | 0.14 | 0.34 | 0 | 1 |
| perceived disorder | 8521 | 1.29 | 0.32 | 1 | 2.11 |
| perceived disorder squared | 8521 | 1.76 | 0.95 | 1 | 4.47 |

* The range of this variable indicates that some respondents reported birthdays of children who are not their own biological children, e.g. when a younger wife lives with an older husband who has children from a previous marriage or relationship.

Source: DFG-Project „Moving for the Kids“, own calculations

# References

Alba, R., Nee, V. (2004): Assimilation und Einwanderung in den USA. In: Bade, Bommes (Hg.) IMIS-Beiträge, Osnabrück

Boudon, R., (1981): The logic of social action: An introduction to sociological analysis. Routledge, Boston.

Breton, R. (1964): Institutional Completeness of Ethnic Communities and the Personal Relations of Immigrants. American Journal of Sociology 70 (2), 193–205.

Ceylan, R. (2006): Ethnische Kolonien. Entstehung, Funktion und Wandel am Beispiel türkischer Moscheen und Cafés. Wiesbaden: VS Verlag für Sozialwissenschaften.

Collier, P. (2013): Exodus: How migration is changing our world. New York : Oxford Un. Pr., 2013.

Crowder, K. (200): The racial context of white mobility: An individual-level assessment of the white flight hypothesis. Social Science Research 29, 223–257.

Dancygier, R.M., Laitin, D.D. (2014): Immigration into Europe: Economic Discrimination, Violence, and Public Policy. Annual Review of Political Science 17 (1), 43–64.

Drever, A. I. (2004): Separate Spaces, Separate Outcomes? Neighbourhood Impacts on Minorities in Germany. Urban Studies 41 (8), 1423-1439.

Esser, H. (2010): Assimilation, Ethnic Stratification, or Selective Acculturation? Recent Theories of the Integration of Immigrants and the Model of Intergenerational Integration. Sociologica 2010 (1), 1–28.

Goyette, K., Iceland, J., Weininger, E. (2014): Moving for the Kids: Examining the Influence of Children on White Residential Segregation. City & Community 13 (2), 158–178.

Greene, W.H., Hensher, D.A., 2010. Modeling ordered choices: A primer. Cambridge University Press

Hanhoerster, H. (2015): Should I stay or should I go? Locational decisions and coping strategies of Turkish homeowners in low-income neighbourhoods, Urban Studies 52 (16), 3106-3122.

Häußermann, H., Siebel, W. (2000): Soziologie des Wohnens. Eine Einführung in Wandel und Ausdifferenzierung des Wohnens. 2., korrigierte Auflage. Weinheim: Juventa-Verlag. ISBN 3-7799-0395-4.

Heitmeyer, W. (1996): Für türkische Jugendliche in Deutschland spielt der Islam eine wichtige Rolle: Erste empirische Studie: 27 Prozent befürworten Gewalt zur Durchsetzung religiöser Ziele. Zeit online (23. August 1996 - 14:00 Uhr).

Horr, A. (2008): Ethnische und soziale Unterschiede der Wohnungssuche und Wohnortwahl. In: Hillmann, Windzio (Hg.) – Migration und städtischer Raum - Chancen und Risiken der Segregation und Integration, Opladen: Budrich, S. 175-192.

Horr, A., Hunkler, C., Kroneberg, C. (2018): Ethnic Discrimination in the German Housing Market. Zeitschrift für Soziologie 47(2), 134-146.

Kabisch, N., Haase, D. (2014): Green justice or just green?: Provision of urban green spaces in Berlin, Germany. Landscape and Urban Planning 122, 129–139. 10.1016/j.landurbplan.2013.11.016.

Lersch, P. M. (2013): Place Stratification or Spatial Assimilation?: Neighbourhood Quality Changes after Residential Mobility for Migrants in Germany. Urban Studies 50 (5), 1011–1029.

Loftus, E.F., Marburger, W. (1983): Since the eruption of Mt. St. Helens, has anyone beaten you up? Improving the accuracy of retrospective reports with landmark events. Memory & Cognition 11, 114–1120.

Massey, Douglas S.; Denton, Nancy A. (1985): Spatial Assimilation as a Socioeconomic Outcome. American Sociological Review 50 (1), 94-106.

Oeltjen, M., Windzio, M. (2019): Räumliche Segregation durch ungleiche Bildungskontexte? Kölner Zeitschrift für Soziologie und Sozialpsychologie 71 (4), 651–675.

Park, R.E., Burgess, E.W., MacKenzie, R.D. (1967): The city. Univ. of Chicago Pr..

Portes, A. (1998): Social capital: Its origins and applications in modern sociology. Annual Review of Sociology 24, 1–24.

Putnam, R.D. (2000): Bowling alone: the collapse and revival of American community. Simon & Schuster, New York, NY.

Quillian, L. (2002): Why Is Black–White Residential Segregation So Persistent?: Evidence on Three Theories from Migration Data. Social Science Research 31 (2), 197–229.

Rossi, P.H. (1954): Why families move, London: Sage.

Sampson, R.J., Raudenbush, S. (2004): Seeing disorder: Neighborhood stigma and the social construction of "Broken Windows". Social Psychology Quarterly 67, 319–342.

Schelling, T.C. (1978): Micromotives and macrobehavior, 1st ed. Norton, New York, 252 pp.

Schönwalder, K., Söhn, J. (2009): Immigrant Settlement Structures in Germany: General Patterns and Urban Levels of Concentration of Major Groups. Urban Studies 46 (7), 1439–1460.

Secchi, D., Herath, G.B. (2019): Parallel society: Myth or reality? A question for policy makers. Journal of Simulation 19 (1), 1–11.

Taeuber, K.E., Taeuber, A.F. (1964): The Negro as an Immigrant Group: Recent Trends in Racial and Ethnic Segregation in Chicago. American Journal of Sociology 69 (4), 374–382.

Taft, R. (1957): A psychological model for the study of social assimilation. Human Relations 10 (2), 141–156.

Teltemann, J., Dabrowski, S., Windzio, M. (2015): Räumliche Segregation von Familien mit Migrationshintergrund in deutschen Großstädten: Wie stark wirkt der sozioökonomische Status? Kölner Zeitschrift für Soziologie und Sozialpsychologie 67, 83–103.

Tibi, B. (2002): Muslim migrants in Europe: Between Euro-Islam and Ghettoization. In: AlSayyad, N., Castells, M. (Eds.) Muslim Europe or Euro-Islam. Politics, culture, and citizenship in the age of globalization. Lexington Books, Lanham, Md, Berkeley, pp. 31–52.

van Parijs, P., 1982. Perverse Effects and Social Contradictions: Analytical Vindication of Dialectics? British Journal of Sociology 33 (4), 589–603.

Wiesemann, Lars (2008): Wohnstandortentscheidungen türkischer Migranten im Vergleich. In: Hillmann, Felicitas / Windzio, Michael (Hrsg.), Migration und städtischer Raum. Chancen und Risiken der Segregation und Integration. Opladen & Farmington Hills: Budrich UniPress, S. 193–211.

Will, A.-K. (2019): The German statistical category “migration background”: Historical roots, revisions and shortcomings. Ethnicities 19 (3), 535–557.

Wimmer, A. (2013): Ethnic boundary making: Institutions, power, networks. Oxford: University Press.

Windzio, M. (2013): Regressionsmodelle für Zustände und Ereignisse: Eine Einführung. Springer VS, Wiesbaden.

Windzio, M. (2016): Children's and adolescents' peer networks and migrant integration. In: Punch, S., Vanderbeck, R. (Eds.) Families, Intergenerationality, and Peer Group Relations. Springer Science + Business Media, Singapore, pp. 1–20.

Windzio, M. (2018): Social exchange and integration into visits-at-home networks: Effects of third-party intervention and residential segregation on boundary-crossing. Rationality and Society 30 (4), 491–513.

Windzio, M., Trommer, M. (2019): Not Just by Walking Distance: Residential Segregation and Children’s Network Integration in the City of Bremen. Urban Affairs Review 36 (1), 1153–1174.

Zelinsky, W. (2001): The enigma of ethnicity: Another American dilemma. Iowa City: University of Iowa City Press.
