# Supplementary material for: Moving for Diversity or Moving for the Kids? The Micro-Dynamics of Residential Relocations During Family Formation of Immigrants and Natives
Source: Front Sociol. 2020 Oct 26;5:538946. doi: 10.3389/fsoc.2020.538946 (PMC8022484; doi:10.3389/fsoc.2020.538946)
Supplement: Supplementary file 2 [file Data_Sheet_1.PDF]

**Moving for diversity of moving for the kids?**
**The micro-dynamics of residential relocations during family formation of immigrants and natives.**

**Michael Windzio[1], Mareike Oeltjen[1], Alice Blanksma[1]**

**[1]SOCIUM, University of Bremen**

Family formation is a crucial event in the life course and generates a major part of residential relocations. After family formation, neighbourhoods become re-evaluated, now as contexts for children's development and socialization. We argue that the perceived or assumed quality of schools and neighbourhoods is an important condition of choosing a destination. However, as the literature on 'ethnic colonies' and immigrant-native residential segregation suggests, immigrants differ from natives in their neighbourhood preferences and relocation patterns. If relocations of migrant and native families to particular destinations do indeed occur basically during family formation and family enhancement, and if they are at the same time outcomes of different preferences, the micro-dynamics of young families' adaptation of housing conditions might have a considerable impact on segregation.

Results of our ordered Heckman probit and event history models show that on the one hand, immigrants and natives tend to different evaluations of characteristics in their neighbourhoods. Particularly respondents of Turkish, Arabic or African origin highly appreciate living nearby a house of worship and also with many non-Germans. On the other hand, our analysis of how these evaluations transform into residential relocations did not show any differences between immigrants and natives. Results thus suggest that evaluations or preferences during family formation do not trigger relocations which result in 'ethnic colonies' at the macro level.

mwindzio@uni-bremen.de

moeltjen@uni-bremen.de

albl@uni-bremen.de
